# Tumor-Like Reaction to Polypropylene Mesh from a Mid-Urethral Sling Material Resembling Giant Cell Tumor of Vagina

**DOI:** 10.1155/2017/6701643

**Published:** 2017-12-18

**Authors:** Ali Azadi, James A. Bradley, Dennis M. O'Connor, Amir Azadi, Donald R. Ostergard

**Affiliations:** ^1^Norton Urogynecology Center, Norton Healthcare, 4001 Dutchmans Lane, Louisville, KY 40207, USA; ^2^University of Louisville School of Medicine, Louisville, KY 40202, USA; ^3^Clinical Pathology Associates, Norton Healthcare, 4001 Dutchmans Lane, Louisville, KY 40207, USA; ^4^UCLA School of Medicine, Los Angeles, CA, USA

## Abstract

**Background:**

Polypropylene material is widely used in gynecological surgery. There are few reports regarding its carcinogenic potential. There is lack of evidence supporting tumor formation directly attributed to the use of polypropylene material.

**Case:**

This patient is a 49-year-old woman with a history of stress urinary incontinence which required a MiniArc® Sling who presented with a hard, tender, immobile mass on the anterior vaginal wall. Pathological analysis of the mass revealed a tumor-like reaction to the polypropylene material that resembled a giant cell tumor of soft tissue.

**Conclusion:**

The use of polypropylene in surgery is ubiquitous across disciplines; thus consideration for a tumor-like reaction to the material should exist for patients who present with a mass near the surgical site.

## 1. Introduction

Giant cell tumor of soft tissue (GCTST) is a rare lesion that has sporadically been reported in the literature as far back as the early 19th century [1, 2]. Extraossseous giant cell tumors have been reported in numerous anatomical sites, such as the breast, head, neck, vulva, and superficial and deep fascia of skeletal muscle [1]. Histologically, GCTST is comparable to its bony counterpart, giant cell tumor of the bone (GCTB), demonstrating a mixture of mononuclear cells with round to oval nuclei and osteoclast-like multinucleated giant cells. Similar to GCTB, the majority of primary GCTST is thought to be benign; however, the metastatic potential of some GCTST lesions has been highlighted in the literature, with the most common site of metastasis being the lungs [3].

Recently, it was reported that a giant cell granuloma grew around polypropylene suture that had been used in a tendon transfer procedure, and the histopathology was consistent with a foreign-body reaction to the polypropylene material. Similarly, there was a case of a suture granuloma that occurred 12 years after an open appendectomy, and several reports in the literature describe foreign-body reactions caused by suture material that mimic cancer [4]. The possible carcinogenicity of polypropylene mesh was noted by the World Health Organization in 1999 following several animal studies; however, its carcinogenicity in humans has not been established. Despite widespread use of polypropylene, there are only a limited number of reported cases to suggest carcinogenicity [5–9].

The presence of foreign bodies is known to induce inflammation, and foreign-body induced inflammation is a recognized factor known to modulate tumor progression [10]. Giant cell tumors of the vagina are extremely rare. To our knowledge, the case that we are reporting is the first case of a tumor-like reaction resembling primary giant cell tumor of the vagina in the English-language literature. A systematic search was conducted on the Pubmed database using the search terms “giant cell tumor soft tissue,” “giant cell tumor vagina,” and “giant cell tumor pelvis.” We report a case of a tumor-like reaction with features of benign GCTST in the vagina associated with polypropylene mesh and discuss the clinicopathological features of this lesion [1].

## 2. Case

**Figure 1:**
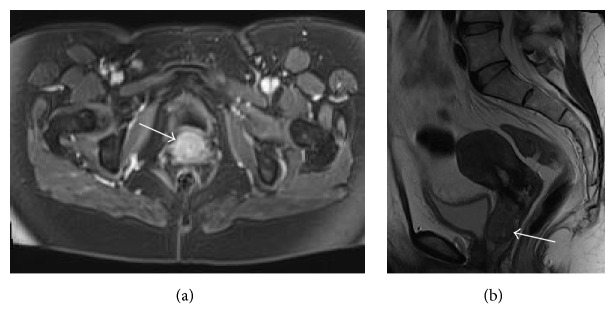
CT scan of the pelvis with (a) and without contrast (b) shows lesion on the anterior wall of the vagina, adjacent to the bladder (denoted by white arrow).

A 49-year-old woman presented to clinic due to pelvic pressure and dyspareunia. Her medical history was significant for Graves' disease and tricuspid regurgitation. Her surgical history was significant for a MiniArc sling for stress urinary incontinence four years prior to presentation, endometrial ablation for abnormal uterine bleeding two years ago, remote C-section, and a laparoscopic bilateral tubal ligation. During examination, a hard, tender, and immobile mass was palpated on the anterior wall of the vagina. The epithelium covering the mass was intact and there was no discharge or bleeding noted during the examination. Due to her complaints of urinary urgency and frequency, cystourethroscopy was initially performed. No abnormality was found in the urethra by evaluation using a 0-degree cystoscope. The intravesical cavity was without any abnormal findings using a 70-degree cystoscope and with complete evaluation of the entire bladder including all edges. An MRI revealed a lesion measuring 3.1 × 2.4 × 2.2 cm shown along the anterior wall of the vagina, adjacent to the base of the bladder (Figure 1).

**Figure 2:**
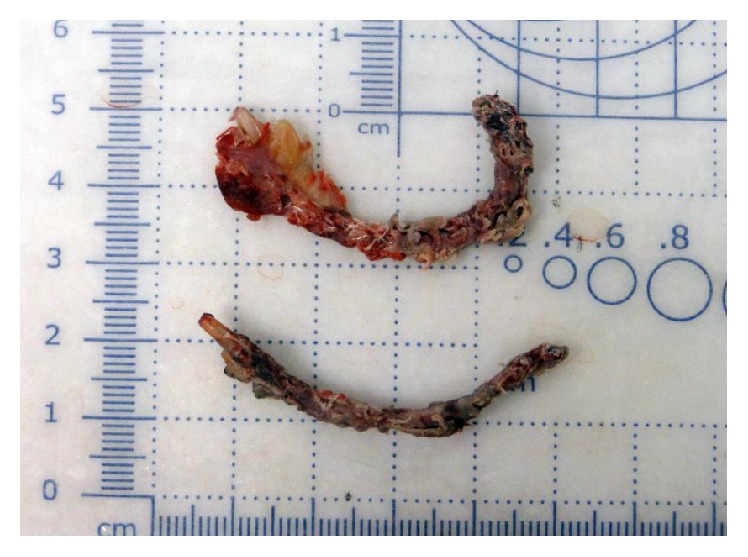
Gross specimen of sling.

The patient opted for removal of the vaginal mesh as dyspareunia had occurred after its placement. Vaginal excision of the mesh and surrounding mass was performed by making a vertical incision in the anterior vaginal wall, and cystourethroscopy confirmed the integrity of the bladder and urethra following the procedure. Specimens of the mesh and surrounding mass were sent to pathology for evaluation (Figure 2). It consisted of red-pink soft tissue measuring 3 × 2.5 cm admixed with blood clot.

**Figure 3:**
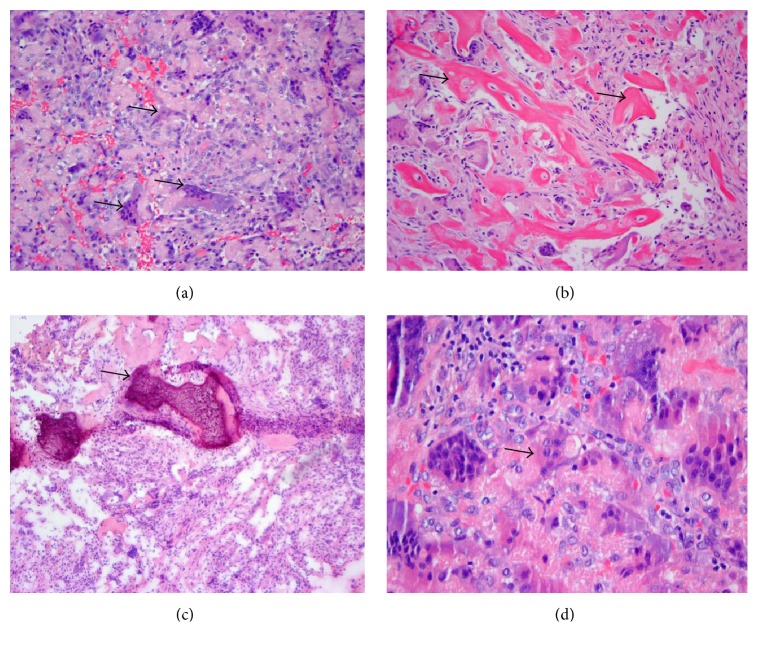
(a) Large numbers of giant cells present in a fibroblastic background (H&E stain-intermediate power; denoted by black arrows). (b) Osteoid-like material in a fibrous background (H&E stain-intermediate power; denoted by black arrows). (c) Dystrophic calcifications (H&E stain-intermediate power; denoted by a black arrow). (d) Ingestion of foreign material by a giant cell (H&E stain-high power; denoted by a black arrow).

Histologically, specimens demonstrated a marked fibroblast reaction with large numbers of giant cells (Figure 3(a)). Many of these giant cells exhibited osteoclastic features. Osteoid-like substance and dystrophic calcifications resembling bone formation were noted in one specimen (Figures 3(b) and 3(c)), and several giant cells were found to surround nonpolarizable foreign material (Figure 3(d)). The proliferative nature of the fibroblasts and giant cells suggested a neoplastic characteristic with benign proliferative reactions, possibly representing a giant cell tumor of soft tissue.

## 3. Discussion

The use of polypropylene mesh for treatment of pelvic organ prolapse and urinary incontinence has increased over the past decade. Some controversy exists within the field regarding appropriate concerns that should be discussed with patients when considering the use of mesh in gynecological surgery. In 2011, the FDA issued a public health notification of adverse events that stated “serious complications with surgical mesh for transvaginal repair of POP are not rare.” Shortly thereafter, on behalf of and endorsed by over 600 members of the Pelvic Surgeons Network, a separate review of the literature highlighted their belief that the FDA presented a biased view regarding vaginal mesh use in all repair procedures for pelvic organ prolapse [11]. Limited data exist regarding the complications of polypropylene mesh in the vagina after the body has been exposed to this material for several decades. A follow-up study of 90 women on the long-term efficacy of the tension-free vaginal tape procedure for stress urinary incontinence showed an objective 90% cure rate after 11.5 years with no adverse effects from the polypropylene tape material or erosion into adjacent tissues [12]. Despite the wide use of polypropylene mesh, there has not been an established relationship between cases of human cancer attributed to the material.

Histologically, GCTST is characterized by a mixture of mononuclear cells and osteoclast-like multinucleated giant cells. Metaplastic bone formation at the periphery of the lesion is observed in 40–50% of cases [1]. Cystic changes and the formation of blood-filled lakes, changes that are similar to aneurysmal bone cystic changes, are present in approximately 30% of tumors. Foci of necrosis are very rare and cytological atypia is absent even if there is a high mitotic activity and vascular invasion. Immunohistochemically, CD68 immunoreactivity is frequently strong and diffuse in the multinucleated giant cells, whereas it is focal in the mononuclear cells. Histopathologically, GCTST should be separated from other tumors which can also exhibit giant cell components such as giant cell tumor of tendon sheath, extraskeletal osteosarcoma, or other benign reactive processes containing abundant osteoclast-like giant cells [1]. Local recurrence has been described after incomplete surgical excision, though metastases, which are characterized by nuclear atypia, pleomorphism, and atypical abundant mitoses, are extremely rare [1].

In a recent case report, recurrence of colon cancer was suspected after a suspicious lesion appeared on CT and PET scans, requiring exploratory laparotomy; a giant cell granuloma had developed around mesh used for prior abdominal hernia repair, demonstrating the ability of these lesions to mimic cancer and unavoidable surgical intervention [4].

In summary, we describe the first case of a tumor-like reaction resembling a primary GCTST in the vagina. Giant cell tumors are rare and likely underrecognized. The foreign-body reaction can make the clinical picture confusing. Consistent with the histology, the lesion in our patient could have developed as a result of the foreign-body reaction to the polypropylene mesh that had been used in the sling. Despite the benign nature of GCTST, formation of any mass can be very stressful for patients, specifically if they have had a prior diagnosis of cancer and are concerned for recurrence. Further research is warranted to better understand the inflammatory reaction to polypropylene due to its widespread use in gynecological procedures and the increased risk that patients may develop a mass late in life as a result of material used during surgery.

## References

[1] Fletcher C. D. M. World Health Organization., and International Agency for Research on Cancer., WHO classification of tumours of soft tissue and bone., , World Health Organization classification of tumours.

[2] O'Connell J. X., Wehrli B. M., Nielsen G. P., Rosenberg A. E. (2000). Giant cell tumors of soft tissue: A clinicopathologic study of 18 benign and malignant tumors. *The American Journal of Surgical Pathology*.

[3] Guccion J. G., Enzinger F. M. (1972). Malignant giant cell tumor of soft parts. An analysis of 32 cases. *Cancer*.

[4] Kassem M. A., Alagiozian-Angelova V., Samuel J. (2010). All that glitters is not gold: Cancer-mimicking lesion in a cancer survivor. *Community Oncology*.

[5] Moller K., Mathes G. L., Fowler W. (2004). Primary leiomyosarcoma of the vagina: A case report involving a TVT allograft. *Gynecologic Oncology*.

[6] Ahuja S., Chappatte O., Thomas M., Cook A. (2011). Bowel cancer and previous mesh surgery. *Journal of Gynecologic Surgery*.

[7] Lin H. Z., Wu F. M., Low J. J. H., Venkateswaran K., Ng R. K. W. (2016). A first reported case of clear cell carcinoma associated with delayed extrusion of midurethral tape. *International Urogynecology Journal and Pelvic Floor Dysfunction*.

[8] Goldman H. B., Dwyer P. L. (2016). Polypropylene mesh slings and cancer: An incidental finding or association?. *International Urogynecology Journal and Pelvic Floor Dysfunction*.

[9] Birolini C., Minossi J. G., Lima C. F., Utiyama E. M., Rasslan S. (2014). Mesh cancer: long-term mesh infection leading to squamous-cell carcinoma of the abdominal wall. *Hernia*.

[10] Klopfleisch R., Jung F. (2017). The pathology of the foreign body reaction against biomaterials. *Journal of Biomedical Materials Research Part A*.

[11] Murphy M., Holzberg A., Van Raalte H., Kohli N., Goldman H. B., Lucente V. (2012). Time to rethink: An evidence-based response from pelvic surgeons to the FDA safety communication: "UPDATE on serious complications associated with transvaginal placement of surgical mesh for pelvic organ prolapse". *International Urogynecology Journal*.

[12] Nilsson C. G., Palva K., Rezapour M., Falconer C. (2008). Eleven years prospective follow-up of the tension-free vaginal tape procedure for treatment of stress urinary incontinence. *International Urogynecology Journal and Pelvic Floor Dysfunction*.

